# Perspectives of Saudi Occupational Therapists Regarding Telerehabilitation: A Qualitative Study

**DOI:** 10.5195/ijt.2023.6584

**Published:** 2023-12-12

**Authors:** Naif Q. Aljabri, Kim Bulkeley, Anne Cusick

**Affiliations:** 1 College of Medical Rehabilitation Sciences, Taibah University, Saudi Arabia; 2 Faculty of Medicine and Health, The University of Sydney, Australia

**Keywords:** e-health, Occupational Therapy, Rehabilitation, Saudi Arabia, Telerehabilitation

## Abstract

**Background:**

Telerehabilitation is emerging in Saudi Arabia. This study investigated occupational therapy professionals' perspectives on using telerehabilitation in their practice.

**Method:**

Data were collected through semi-structured phone interviews conducted with nine Saudi occupational therapists. A pragmatic qualitative evaluation approach was used.

**Findings:**

Experience and perceptions of participants regarding telerehabilitation were represented as follows: awareness and knowledge of telerehabilitation; how telerehabilitation increases occupational therapy availability and access in Saudi Arabia; telerehabilitation in the pandemic; telerehabilitation is preferred; suitability of telerehabilitation in Saudi Arabia; telerehabilitation care pathways; telerehabilitation readiness in Saudi Arabia; and telerehabilitation willingness by Saudi occupational therapists.

**Conclusion:**

Saudi occupational therapists have good knowledge and awareness of telerehabilitation, and some had used it during the pandemic. They showed positive attitudes and a willingness to use telerehabilitation if appropriate technology infrastructure, official policy standards and guidelines, training, data security, and financial resources could be provided to support implementation.

Telerehabilitation enables patients to receive occupational therapy (OT) services within their homes or local environments without needing to travel to service sites. In the Middle East and North Africa (MENA) region, technology-enabled rehabilitation can remove barriers to service access including transportation infrastructure, distance and centralisation of rehabilitation services to cities (Aljabri et al., 2021). The utility of telerehabilitation in opening-up access was clearly demonstrated in the COVID-19 pandemic in 2020, when globally the focus on delivering health services unexpectedly shifted from in-person to e-health and telerehabilitation (Garfan et al., 2021).

In Saudi Arabia, OT is provided in hospitals across different health sectors including: the Ministry of Health hospitals, National Guard Health Affairs hospitals, Aramco Healthcare, Armed Forces hospitals, Security Forces hospitals, Royal Commission hospitals and teaching and university hospitals (Ministry of Health, 2018). These services are concentrated in cities with scant rehabilitation availability outside central metropolitan areas. There is a demonstrated workforce shortage and need for more occupational therapists (OTs) (Alshehri et al., 2019). During COVID-19, OT and rehabilitation services were disrupted (Almubark et al., 2022; Ilyas et al., 2021) in Saudi Arabia with movement restrictions and local and nationwide lockdowns (Almubark et al., 2022). In response, telerehabilitation was successfully implemented in some settings in Saudi Arabia with positive patient and provider acceptability (Aljabri et al., 2021; Alrushud et al., 2022; Alsobayel et al., 2021).

Even before the pandemic, Saudi Arabia's “Vision 2030” recognised e-health services as a legitimate part of health care delivery (Ministry of Health, 2019; 2020). But in OT practice in Saudi Arabia, little is known about using telerehabilitation from the perspective of OTs. To date, no study has investigated OTs' knowledge, attitudes, and perspectives about using telerehabilitation in their practice in Saudi Arabia. While evidence relating to this may be available for OTs in other countries or for other health professionals in Saudi Arabia, nothing is known about the Saudi OT experience. Localised evidence is needed to appropriately identify workforce needs and implementation issues should the extension of OT telerehabilitation services in Saudi Arabia be needed and desirable. This study thus aimed to investigate OTs' perspectives about telerehabilitation, including attitudes, perceived enablers, and barriers to implementation in practice, either as existing or future telerehabilitation users and providers.

We had three research questions: (1) What do Saudi OTs know about telerehabilitation? (2) What perspectives do Saudi OTs have about using telerehabilitation, including their attitudes, views about enablers and barriers to implementation, and any other issues they perceive regarding the implementation of telerehabilitation in OT practice? (3) What are Saudi OTs' perspectives on telerehabilitation in their practice based on their experience or observations during the COVID-19 pandemic?

## Method

A qualitative research design using a pragmatism approach was used to enquire into the experience and perceptions of OTs regarding telerehabilitation in Saudi Arabia (Kaushik & Walsh, 2019; Morgan, 2014; Patton, 2014). This approach was selected because it permits a utilization focus within service settings.

The study used semi-structured interviews with volunteer OTs who had practice experience in Saudi Arabia and were Saudi citizens. To recruit, information about studies being conducted by the team on the topic of telerehabilitation in Saudi Arabia was distributed by email to members of the Saudi OT Association. This information was in English. This is because all OTs in Saudi Arabia's health care sectors can read, speak, and write in English because all OT programs in Saudi Arabia are delivered in English. English was also the common language of the research team. People who were interested in being interviewed provided their contact details via a secure electronic data capture platform called Redcap (Research Electronic Data Capture, Harris et al., 2009) hosted at the University of Sydney. They were informed that submitting their contact details would be considered consent to participate as volunteers. Potential participants were then emailed by the first author to confirm eligibility and to provide them with a detailed participant information statement so that they could make an informed decision about whether or not to proceed.

Interviews could be conducted in Arabic or English as preferred by participants. All chose Arabic which was their parent language and also the first language of the interviewer (NA). In Saudi Arabia, gender segregation is a cultural practice (Alsadaan et al., 2021). For this reason, phone interviews were selected as the data collection method. Twenty-two people expressed interest, with nine then agreeing to participate as volunteers. Interview arrangements were agreed in advance; participants knew the interview would be audio-recorded and then transcribed with identifying information removed.

An interview question-and-prompts guide was used. The study's purpose was explained again, referring back to the participant information statement previously sent. The interviewer restated the voluntary nature of the interview and noted participants could withdraw at any time during the interview. An undertaking was made that the identity of volunteers would remain confidential and that every effort would be made when reporting findings to ensure anonymity. The remainder of the interview guide comprised questions and prompts related to issues about telerehabilitation in practice revealed in a literature review study previously completed by the team (Aljabri et al., 2021). Although the interview was in Arabic, English words and phrases were also used by participants.

Interviews were digitally recorded then professionally transcribed. The Arabic transcriptions were inspected by the first author for accuracy in terminology used by the transcription service. Identifying information was removed at this point before transcriptions were emailed to individual participants for checking, additions or amendments and approval for use. Seven of the transcripts were approved without alteration; two had some amendments by participants prior to approval. To permit collaborative coding and analysis, two full transcripts were professionally translated from Arabic to English and then back-translated from English to Arabic. Only two were translated because of the cost for professional translation services. Professional translation of at least some of the data was considered an important feature of Arabic-English qualitative research as Aloudah describes (Aloudah et al., 2018). The professionally translated English transcripts were used by the team for thematic analysis and development of the initial coding frame (Saldana, 2021). The other seven transcripts had excerpts translated into English by the first author who is fluent in both English and Arabic to support the analysis process. All transcripts were discussed in analysis meetings in English with excerpts used to illustrate.

A thematic analysis strategy was used to identify, analyse, and report themes and patterns within the data, allowing the author(s) to focus on recognising and characterising overt and covert concepts present within the data (Braun & Clarke, 2006). A combination of inductive and deductive analysis approaches was employed to explore the use of telerehabilitation in OT practice in Saudi Arabia (Azungah, 2018). Initially inductive coding was used to create meaning-unit codes by reviewing transcripts line-by-line. Deductive coding was then used to cluster codes and identify themes and categories. We used NVivo software (Release 1.6.1) to support the analysis and coding of the professionally translated English interviews. But because NVivo does not support the Arabic language, the remaining data entered into NVivo came from researcher-translated quotes, selected for their relevance to existing codes or new ones in consultation in the research team. This approach to analysis of translated quotes for codes has been recommended as a suitable way to develop key themes and categories in Arabic translation data (Alodhayani et al., 2021).

To familiarise ourselves with data, findings were initially organised according to the type of information provided in response to specific questions. Responses provided information about: what they knew about telerehabilitation and what they saw as benefits; if they had experience with telerehabilitation during COVID-19 lock-downs; examples of their telerehabilitation experience; when they thought it appropriate to use telerehabilitation; how it might be done; what was needed for implementation (training, infrastructure); and challenges regarding telerehabilitation implementation. The data for these topic-categories was discussed by the team with researcher-translated quotes having special attention to ensure clarity, consistency, and relevance in relation to the code. These topic-categories were then further interrogated (by NA and AC) to reveal the standpoint of, and processes used by participants in their perceptions and lived experience of telerehabilitation. These are the thematic categories presented in the results – they describe and explain perceptions and experience in relation to telerehabilitation. Once codes were finalized, deductive analysis for themes and categories was shared with the remaining research team member (KB) and this occurred in English. Findings were reported as narrative synthesis.

## Results

Nine Saudi OTs (three females; six males) participated in interviews that ranged from 20 to 47 minutes. To ensure anonymity in reporting no age was disclosed, all data relating to participant characteristics was aggregated and de-identified (e.g., not using the name of a city) and not linked to specific quotes from individuals. Any combination of these characteristics could potentially identify an individual, since the OT workforce in Saudi Arabia is limited (World Federation of Occupational Therapists, 2020). Highest qualifications were: four postgraduate (level not identified to maintain anonymity) and five undergraduate. All worked in metropolitan cities at hospitals (Ministry of Health: university-teaching hospitals and clinics: military hospitals).

Experiences and perceptions of participants regarding telerehabilitation were captured in eight thematic categories: awareness and knowledge of telerehabilitation; how telerehabilitation increases OT availability and access in Saudi Arabia; telerehabilitation in the pandemic; telerehabilitation is preferred; suitability of telerehabilitation in Saudi Arabia; telerehabilitation care pathways; telerehabilitation readiness in Saudi Arabia; and telerehabilitation willingness by Saudi OTs. These are now presented with supporting data.

### Awareness and Knowledge of Telerehabilitation

All participants were aware of telerehabilitation as a mode of practice used by health professionals in Saudi Arabia and elsewhere. Their knowledge of what telerehabilitation was, is accurate with reflecting key attributes found in literature. This is an example: “…*telerehabilitation is any method a therapist uses to treat a patient or client in a non-in-person way or not directly in front of him…This includes social [media] applications, such as WhatsApp or FaceTime*.” (P4). Here is another example that does not refer to specific information technology (IT) applications: “*I heard that telerehabilitation means using some technological devices to assist the patient remotely, not in-person service with them*.” (P5). Another way they expressed awareness of telerehabilitation was to use names of local services – for example, “…*the virtual clinic is what we call telerehabilitation or these terms that we use, but the most widely used is the virtual clinic*.” (P9).

### How Telerehabilitation Increases OT Availability and Access in Saudi Arabia

A physician referral is required for OT services in Saudi Arabia. Once received, appointments with OTs need to be arranged. Depending on the service and site there can be waiting lists or delays for weeks or months: “*Patients are referred to the occupational therapy department by a physician…It takes a long time for an occupational therapy appointment*…” (P6). Multidisciplinary teamwork is not common in Saudi hospitals, so a referral will be the first time an OT department will know a new patient requires service. When demands for service are higher than available OT caseload-hours, a patient may be discharged before the OT practitioner can see them, or before treatment can be implemented after an initial assessment. In these situations, telerehabilitation can make OT available to Saudi patients when otherwise it would not be. For example, “*Sometimes we have a workload and overload of patients, so telerehabilitation will make this matter easier for them and us*.” (P3). Telerehabilitation thus ensures the physician referral can be implemented to ensure care as it was prescribed.

In addition to caseload management, telerehabilitation was identified as an effective means to provide accessible OT to Saudi patients in post-discharge or outpatient settings where in-person attendance is not feasible. Geographical distance, for example, can be a barrier especially when rehabilitation services are centralized in hospitals and community-based rehabilitation opportunities are scant, “…*We have many patients who live outside the city, so it is difficult for them to come to us sometimes*.” (P3). Other factors may impact a patient's ability to come in-person to outpatient OT such as travel expenses, time needed by family members to accompany the patient, inability for the patient to travel alone. In these cases, “*If the patient cannot come to the hospital, telerehabilitation eases accessibility*.” (P2).

### Telerehabilitation in the Pandemic

Saudi Arabia implemented public health measures restricting movement and in-person services as a response to the COVID-19 pandemic (Ministry of Health, 2020). OTs who previously only ever provided in-person service either had to pivot to alternative delivery modes such as e-health/telerehabilitation or suspend service. It was at this time, ‘telerehabilitation’ became more widely known, for example: “*I started hearing about this thing only during the period of COVID-19, but before that, I did not know about it*…” (P5) and “*We didn't understand the actual perception of telerehabilitation; we didn't understand it before the pandemic, but we do now*.” (P9).

For four of the participants, telerehabilitation became part of their everyday practice during the COVID-19 public health restrictions. These therapists worked in hospitals where e-health approaches to service delivery were encouraged: “*The outpatient clinics stopped entirely due to the COVID-19 pandemic…and we had to contact patients…We were given an introductory information about telerehabilitation in the clinic*…” (P9) and “*In the first step, we called all patients individually and explained the reason for that and the method*…” (P5). The therapists were using telerehabilitation for the first time with their patients, “*It was a compulsory experience at the time of the quarantine when the patients were forbidden to roam*…” (P2).

The types of services offered by OTs during COVID-19 pandemic restrictions included follow-up and consultations: “…*we used the phone call only…for follow-ups and if there are consultations* …” (P8) and “…*For example, ‘How are you?’; ‘How are you finding the exercises*?…” (P2).

The shift to telerehabilitation due to the pandemic was sudden and required effort. This participant's response captures the effort needed: “*This step did not exist before COVID-19, not only for patients and us as therapists. Honestly, we are not used to working this way, so it was new to us…to be honest, the matter was not easy*.” (P5). Another therapist received information about using telerehabilitation as a new method of service delivery: “…*We were given an introductory information about telerehabilitation in the clinic. They told us what we could do for patients and how we could do telerehabilitation in the clinic*…” (P9).

Saudi OTs needed to innovate and experiment in order to shift services to a telerehabilitation mode. Some conducted sessions on technology platforms available in the hospital and the home, for example, “*We often used programs such as Zoom, which are available and easy to use even for patients*…” (P5) or “… *the TEAMS program was installed [at the hospital], which is the direct communication program between the patients and us*…” (P9). Others did not have access to hospital-owned or managed devices and technology and instead used their own. For example, phone calls or video calls, “*We used WhatsApp mostly; we have a list of patients and their phone numbers. So, we communicate with them*…” (P2). Some participants were pleased about successful telerehabilitation use during COVID-19: “*The telerehabilitation service was relatively good for me because I explained to the patient via video how to perform the exercise; the patient performed the exercise with me*…” (P9).

### Telerehabilitation is Preferred

Since the pandemic, there has been increased awareness of e-health and telerehabilitation by both staff and patients in Saudi Arabia. Those therapists who used telerehabilitation during the pandemic restrictions have continued to include it as a service delivery option. For example, “*[we can] see more cases, we know how to work because telerehabilitation with some patients is more effective and saves us more effort and time*.” (P5). For patients, acceptance of using technology in receiving therapy increased as therapists noticed: “… *after trying telerehabilitation, the patients who did so considered it feasible and helpful*.” (P8).

Therapists who did not use it during pandemic restrictions now have it on their radar as Saudi patients and other Saudi health professionals suggest it as a preferred mode. For example, “*Some patients came to me after the COVID-19 lockdown, and they asked me to give them the follow-up session via telerehabilitation*…” (P8). Therefore, even after easing COVID-19 restrictions, some Saudi hospitals allowed the therapists to continue to use telerehabilitation, “… *the option of telerehabilitation is still available to us in the hospital…they told us that it is possible to refer some patients to telerehabilitation sessions*.” (P9).

### Suitability of Telerehabilitation in Saudi Arabia

Saudi OTs have views about the suitability of telerehabilitation across caseloads and session-types. They said patient preference may be important in determining when telerehabilitation is used if caseload conditions permit, for example: “… *patients with moderate or fewer health conditions issues may prefer this method*.” (P5). They also suggested therapist assessment of suitability may be required in some caseloads, for example: “*It is suitable for some stable cases that do not need hands-on treatment from a therapist*…” (P9).

Telerehabilitation was particularly suitable for therapy sessions that involved caregivers or the family. For example, “…*the elderly…they can communicate with us, but sometimes we may need to talk to their relatives. So, we gave them instructions [via telerehabilitation] to do with their elderly patient*…” (P8). The opportunity for caregiver involvement was a positive for patients as well, “*They were satisfied that the caregiver attended*.” (P5).

### Telerehabilitation Care-Pathways

Participants conveyed preferences regarding the most appropriate times in a care-pathway for telerehabilitation and they also made suggestions for best ways to implement this.

Participants recommended a patient-centred educational process to introduce not only telerehabilitation but also OT. In-person clinic visits establish rapport, assess patients, and explain telerehabilitation. This may improve the therapeutic relationship and patient confidence in telerehabilitation. For example, “*In the first session, the patient attends to the in-person clinic…, teach him about occupational therapy*…” (P8), and “…*knowledge of patients about the importance of occupational therapy…and what we will offer*…” (P6). Some procedures may need to be completed in-person in that first session, “…*we must first do a physical assessment*…” (P3).

After learning about OT and completing any essential in-person procedures, patients could then be educated about telerehabilitation: “*Patients need to understand the concept of telerehabilitation. Why do we use this service? He needs to have awareness…to be more comfortable; then, we can start using telerehabilitation because the patient's confidence in us is greater*.” (P5).

To progress telerehabilitation to implementation, participants recommended going beyond talking about it with patients and instead, where possible, giving patients exposure to a session whilst still at the in-person clinic. This provides an opportunity for training in skills needed, and it gives patients an immersive experience to develop a complete picture of the nature of using telerehabilitation in receiving OT services. For example, “*It is possible to try this service directly if the patient does not have an understanding or background about the service, which is possible during his visit*.” (P7).

In relation to care-pathways, telerehabilitation was viewed as most suitable following hospital discharge; for example, “… *follow-up can be done while the patient is at home using telerehabilitation*…” (P8). It was a useful option to consider if the purpose of the service was specifically to check in on the progress of patient, “…*It can be helpful as follow-up or as a consultation only*” (P3).

Preferred IT for telerehabilitation in OT services were video call methods via any available devices with their patients; “*I will use video calling; I prefer that it always be a camera via computer…using voice [call] can cause a misunderstanding between the patient and the therapist*.” (P1). In addition, video calls permit non-verbal instruction, “…*some patients need you to be standing and talk and make the movements that you ask of them*…” (P4).

### Telerehabilitation Readiness in Saudi Arabia

Participants identified professional development and infrastructure requirements needed to be ready for implementation of telerehabilitation in Saudi Arabia. They wanted training in telerehabilitation practice in general, for example: “*Providing training courses for the use of telerehabilitation; presenting to us how to use telerehabilitation with patients; if someone explains to us how to use programs*…” (P8). In addition, they wanted topic-specific education. Two topics were identified as important. The first was training in the use of ‘health-apps’ for informed selection and use, “… *It would be better if it were easier or at least do a training session for using the program and ensure it is practical*.” (P7). The second was training in practices that would maintain ethics and professional standards such as record-keeping and confidentiality in telerehabilitation, especially when there may be no local guidelines in place. For example, “…*Explain to us if most virtual or phone sessions are recorded, if there is a violation of privacy*…” (P9).

The need for consistent telerehabilitation practice standards was identified in relation to quality of care and in supporting the introduction of this innovative service delivery mode. For example, “*If there or they unified the guidelines…something official, it will make all patients and all authorities feel more at ease. They specify the type of patient and how many sessions and, after that, use tele-rehabilitation*.” (P1).

Readiness for telerehabilitation implementation in Saudi Arabia also required policy, training, and infrastructure, with hospitals needing built and IT resources for OT use. First, telerehabilitation sessions require appropriate venues: a quiet, isolated room to ensure patient privacy and clear communication while online. One therapist stated: “*I want the place to be isolated and quiet*.” (P1). This is crucial if the patient is practicing skills in a treatment session, “…*you need a private room to show the patient what you want him to do between you and him*.” (P2). Second, IT must also be adequate to the task and available to rehabilitation staff. This may require financial investment by hospitals which could be challenging, “*There will be issue with the financial budget*.” (P7).

If such investment was made, updating, or refreshing existing IT may then be needed. As this participant describes: “…*we need to renew or refurbish the devices we have; all our computers are ancient…We also need devices dedicated only to telerehabilitation, such as iPads and tablets in general, or mobile phone devices for this purpose*.” (P4). Additional IT may also be required, for example, “*There are only the usual computers, we do not have the capabilities of a camera*.” (P7).

IT hardware and software needs regular maintenance. Participants described instances during the COVID-19 lockdown period when attempts to provide OT telerehabilitation for their Saudi patients were thwarted by IT that was available but not functioning. As one participant identified, “… *periodic maintenance of the devices, meaning that it is in periodic maintenance because, actually, [if it is not maintained] we suffer from problems with it*.” (P5).

Infrastructure beyond the hospital is also important for effective telerehabilitation. A hospital may have sufficient well-maintained IT, but the capacity for telerehabilitation services and the type of service possible will depend on the speed and consistency of internet connectivity: “…*and the most important thing is high-quality Internet*.” (P4).

One participant recommended dedicated spaces where connectivity and speed can be assured: “*Allocate a specific place where there is good internet*…” (P3), The challenge of internet speed and connectivity is particularly important in Saudi Arabia when patients live in rural and remote settings. For example, “…*in faraway areas from the city; the internet is weak. So, we cannot communicate well with the patient*.” (P4). Sometimes the issue is having internet available at all, for example “*Some patients live outside the city, for example, in governorates or villages. The internet is not at the required speed or unavailable*…” (P8).

When up-to-date and functioning IT is available, telerehabilitation is not only more feasible, but it also conveys a message to staff that it is a supported platform for service delivery. If telerehabilitation is supported with infrastructure, this may also smooth the way for telerehabilitation readiness. For example, “… *The presence of technology, the presence of developed computers, the presence of cameras, all devices and communications are equipped, so I see that the workplace is well-equipped*.” (P6).

### Telerehabilitation Willingness by Saudi OTs

Participants shared their own willingness to use telerehabilitation and their insights regarding using it in their practice. Participants either had used telerehabilitation or they were interested and willing to use it in the future if they had training, access to resources and it was permitted by their hospitals, “*There is no need other than approval, training*…” (P7).

When participants thought about other OTs willingness to use telerehabilitation, there was concern that others may not understand what it is, or they may hold attitudes towards IT that are not positive. For example, “*Unfortunately, some therapists do not like to use technology either, and they think that the use of technology is ineffective*” (P8). Another reason participants thought other OTs may be reluctant to use telerehabilitation was because it was not a supported or approved service delivery method at their place of employment. For example, “*There was no permission for the rehabilitation department to use it and have a remote session*.” (P6). It was noted this situation can change, as happened during the COVID-19 lockdown period when OTs advocated for use of this service delivery method which was then endorsed, “…*We discussed it with the head of the department and decided that we should use telerehabilitation because the quarantine period was long*.” (P8).

Reasons for variation in patient willingness to use telerehabilitation were described. In the first instance, adequate orientation and education about telerehabilitation was needed to ensure patients were ready and able to make informed choices. This preparation was identified in “Telerehabilitation readiness”. A second factor impacting patient readiness is patient access to and comfort in using IT devices. For example, telerehabilitation is less accepted: “*If the patient is elderly or does not know how to use the devices*.” (P1). A third factor perceived by therapists to influence Saudi patient willingness to engage in telerehabilitation is the patients' belief in the effectiveness of the delivery method. For example, “*The main factor that I noticed with the elderly is that they do not accept, do not believe in telerehabilitation, and do not expect that they will have results such as direct in-person therapy sessions*.” (P5). Further, patients may perceive the telerehabilitation approach is less valuable and valuing of them as people – for example, “*Patient feels that I do not want to give him a therapy session*.” (P2). Patients may also be concerned about privacy or the use of IT to record aspects of their performance or condition, for example, “…*they do not want anyone to take a picture or clip so that they may refuse video calls*.” (P1).

## Discussion

This study explored Saudi OTs' experiences and perceptions of telerehabilitation. It provided novel evidence regarding awareness, knowledge, and experience of telerehabilitation in Saudi Arabia from a participant perspective. Localised knowledge is essential to understanding the needs and implementation issues faced by health professionals wanting and needing to integrate and implement new modes of service delivery in their practice. The OTs had good awareness and accurate understanding of what telerehabilitation was and how it could and did increase access to OT services in Saudi Arabia. Some had used telerehabilitation during the pandemic or were made aware of it as a service delivery option. They indicated positive attitudes towards telerehabilitation, a sense of willingness to use it and plans to use it in their practice. These findings are similar to previous research regarding perceptions and experience of other rehabilitation professionals in MENA (Aljabri et al., 2021) and physiotherapy practitioners in Saudi Arabia (Alrushud et al., 2022).

These findings are similar to previous cross-sectional studies regarding the perceptions, attitudes, and experiences of telerehabilitation with other rehabilitation professionals in Saudi Arabia (Aloyuni et al., 2020; Alrushud et al., 2022; Ullah et al., 2021). These studies also reported similar findings on barriers to a financial budget, privacy concerns, technology infrastructure, and providers' skills in using telerehabilitation (Aloyuni et al., 2020; Ullah et al., 2021). Cultural issues did not come up in our study. However, Alrushud et al. (2022) reported variations in Saudi accents nationwide when patients and therapists use telerehabilitation sessions (Alrushud et al., 2022). Therefore, this study reveals that introducing OT services via telerehabilitation at in-person clinic visits will ease communication between therapists and patients before starting the actual telerehabilitation sessions and improve therapeutic relationships (Aljabri et al., 2021). However, our in-depth study includes information that can help in coping with and solving the challenges of telerehabilitation in Saudi Arabia by providing the requirements of building a telerehabilitation clinic, provider training processes, procedures for introducing telerehabilitation for patients and their caregivers and enhancing guidelines and policies for using telerehabilitation from the viewpoints and experiences of OT service providers in the Saudi context to assist care delivery as planned in the ongoing Saudi Vision 2030 (Ministry of Health, 2019; 2020).

Saudi OTs believe and have lived experience of telerehabilitation enabling increased access to OT services. Examples were given of OTs discussing the use of telerehabilitation to maintain service during pandemic lockdowns, implementing telerehabilitation to ensure access when patients could not come to the hospital, and being asked by patients to offer their service via telerehabilitation. This evidence supports the notion that Saudi access to the limited OT workforce could enhance availability in Saudi Arabia (Almubark et al., 2022; Alshehri et al., 2019). Telerehabilitation also reduced time delays in the referral process, travel costs for patients and staff, and pre-and post-session time for patients and therapists that had been previously reported (Aljabri et al., 2021). It was encouraging that participants were willing to use telerehabilitation to make OT services more accessible to patients and they were willing to experiment with IT platforms, venues, and OT activities that could be done online. These findings were consistent with two studies in Kuwait and Saudi Arabia that found that physiotherapists were willing to use telerehabilitation to improve patient access to rehabilitation services (Albahrouh & Buabbas, 2021; Alrushud et al., 2022).

Most participants had experience using telerehabilitation with different technological devices and methods during the COVID-19 pandemic. Although the use of telerehabilitation in providing Saudi rehabilitation services during COVID-19 was sudden, OTs gained valuable knowledge relevant to longer-term feasibility of this mode of delivery; similar experiences were reported in a study in Spain (Báez-Suárez et al., 2022). Participants' experiences also provide evidence of the Saudi infrastructure needed for telerehabilitation implementation including venues, IT, and infrastructure such as high-speed internet. If system-wide implementation of telerehabilitation is desired, these challenges will need to be addressed in Saudi Arabia.

Telerehabilitation usually relies on technology that is readily available to most people (phones, tablets, or computers), participants revealed this technology was not always available within their services. Even if it was, therapists wanted specific training in how to provide therapy via these technologies. Providing training will ensure effective and feasible care (Aljabri et al., 2021; Fakolade et al., 2017). Participants indicated this training should go beyond the use of technology and include education relating to practice behaviours for maintenance of ethical and professional standards in the Saudi Arabia context, for example data confidentiality and appropriate information-sharing processes. These practice behaviours require enabling frameworks such as specific policy guidelines and training processes for both the OTs and their patients (Fakolade et al., 2017; Qureshi et al., 2021). Such training and policy development is consistent with Saudi Arabia's advancement of digital technology in health policies (Ministry of Health, 2019).

Overall, this study presents a promising prospect of a group of informed, willing, and experienced OTs who have an intention to continue using telerehabilitation at the same time as seeing the challenges of sustained and wider use. Therefore, this study is necessary to improve the accessibility of OT services and promote technology and innovation policies and guidelines in Saudi Arabia for improving healthcare delivery, which is aligned with Vision 2030 for the health sector in Saudi Arabia (Ministry of Health, 2019; 2020). Further research is needed regarding implementation to assess and determine the most effective ways of using telerehabilitation among patients, caregivers, healthcare professionals, and health authority decision-makers. Future research could also investigate how best to train and provide continuing professional development support for OTs interested in commencing or sustaining telerehabilitation.

## Study Limitations

The sample was small (N = 9) and restricted to Saudi OTs working in metropolitan hospitals in four health sectors. Despite this study using purposeful sampling, we relied on volunteers who self-selected, so not all types of OTs working in Saudi Arabia were included. These sampling issues could limit the transferability of findings (Silverman, 2021). Another methodological limitation was the ‘insider’ status of the first author – a Saudi OT with similar background to the interviewees (both culturally and professionally). This has been suggested to be a weakness (Roulston & Choi, 2018). The interviewer was male which may also have impacted willingness of potential female participants to join the study even though a phone interview method was selected. Finally, only two of the transcribed interviews were translated and back-translated. The remainder were used in Arabic with excerpts selected and translated into English in consultation with the research team. We recognise that translation is necessarily interpretive, so it is possible that the original meaning may have changed during the translation process (Aloudah, 2022; van Nes et al., 2010).

## Conclusion

Telerehabilitation has potential for more widespread use by Saudi OTs with appropriate technology infrastructure, enabling policy frameworks including privacy, data security, practice behaviours, practice standards and guidelines, training and continuing professional development, and financial resources to support these initiatives. The expanded use of this approach shows great promise in extending the reach of the limited OT workforce resources in Saudi Arabia.
